# Age-stratified risk factors and predictive models for progression to lupus nephritis in patients with systemic lupus erythematosus: a review

**DOI:** 10.3389/fimmu.2025.1682280

**Published:** 2025-11-05

**Authors:** Shuyu Sun, Song Li, Xin Chang, Jian Wu

**Affiliations:** Department of Rheumatology and Immunology, The First Affiliated Hospital of Soochow University, Suzhou, China

**Keywords:** systemic lupus erythematosus, lupus nephritis, age stratification, risk factors, clinical heterogeneity, predictive models

## Abstract

Systemic lupus erythematosus (SLE) is a highly heterogeneous autoimmune disorder, and lupus nephritis (LN) is one of its most severe organ manifestations. The age at disease onset is a critical factor influencing the clinical phenotype, disease progression, and prognosis of SLE. However, few studies have specifically focused on the age-stratified risk of developing LN. This review examines the age-related clinical and immunological features of SLE and the risk factors associated with progression to LN. In addition, it systematically evaluates how current LN risk prediction models incorporate age as a variable. Although many existing models include age, a significant gap remains-no tools have been specifically designed to assess LN risk across different age groups. Therefore, developing age-specific LN risk prediction models and tailored management strategies is crucial to improving patient outcomes. Such approaches would enable the early identification of high-risk patients and facilitate individualized interventions, ultimately leading to improved long-term renal outcomes for patients with SLE.

## Introduction

Systemic lupus erythematosus (SLE) is a chronic autoimmune disease with a complex etiology and diverse clinical manifestations. It affects approximately 3.4 million individuals globally, over 90% of whom are female (1, 2). The incidence and prevalence of SLE vary significantly across regions and ethnic groups (1). SLE is highly heterogeneous in its clinical presentation, potentially involving various organs including the skin, joints, kidneys, blood, and nervous system. Patients demonstrate considerable variability in disease manifestations, progression, and response to treatment (3). This heterogeneity is attributable to multiple factors such as genetic background, immune mechanisms, and age of onset. Collectively, these factors pose significant challenges to clinical classification and the development of personalized treatment strategies.

Lupus nephritis (LN) is one of the most common and severe organ manifestations of SLE. It affects approximately 40% of SLE patients over the course of the disease. In about one-third of cases, LN is the initial clinical manifestation of SLE (4). LN profoundly affects patient survival and quality of life, and it is a leading cause of SLE-related mortality and progression to end-stage renal disease (ESRD) (5, 6). Notably, patients with SLE of Hispanic, African, or Asian descent are more likely to develop a highly active, relapsing form of nephritis with rapid renal function deterioration (4, 7). The course of LN is complex and variable. Despite recent therapeutic advances, including the introduction of novel immunosuppressants such as belimumab, approximately 10-30% of patients progress to ESRD within 10 years, ultimately requiring dialysis or kidney transplantation (4, 8). Furthermore, the long-term use of glucocorticoids and cyclophosphamide (among other immunosuppressants) increases the risk of infections, osteoporosis, and other complications. These adverse effects negatively impact treatment adherence and long-term outcomes (9). Therefore, the development of LN marks a critical turning point in the prognosis of patients with SLE and remains a key focus of clinical management.

SLE can occur at any age, and studies have shown that the age at disease onset strongly influences the clinical presentation, pattern of organ involvement, and overall disease activity. Late-onset SLE (lSLE, diagnosed at 50 years or older) is generally associated with lower disease activity and less extensive systemic involvement, especially manifesting as milder symptoms in the skin, kidneys, and nervous system (10). However, lSLE patients are more likely to accumulate severe chronic organ damage and face a higher risk of mortality (11). In contrast, early-onset SLE (eSLE,diagnosed before about 50 years of age) tends to present with higher disease activity and multi-organ involvement (12, 13). Despite these differences, relatively few studies have systematically examined the age-stratified risk factors, clinical features, and outcome differences in SLE patients who progress to LN. Therefore, this review addresses age stratification in the progression of SLE to LN and summarizes current age-related LN risk prediction models. The goal is to provide a theoretical foundation and practical guidance for the early identification of high-risk patients and the implementation of targeted interventions.

## Age-related clinical and immunological heterogeneity in SLE

The age at onset is recognized as a crucial factor influencing the disease phenotype, immunological features, organ involvement, and disease progression in SLE (12). Although no universally accepted standard for age-based stratification of SLE exists, a common approach categorizes SLE cases into juvenile-onset SLE (jSLE, diagnosed before 18 years of age), adult-onset SLE (aSLE, diagnosed between 18 and 50 years, and lSLE (14). Some studies use 50 years as the cutoff to distinguish eSLE from lSLE, while others define “very-late-onset SLE” (vlSLE) as diagnosis at 60 years or older (15, 16). These varying criteria can affect the comparability of research findings; nevertheless, most studies support a strong association between age at onset and the heterogeneity of SLE.

Patients with jSLE typically exhibit higher disease activity, more characteristic immunological abnormalities, and more extensive organ involvement. However, they are also more likely to show clinical improvement following initial treatment (17). A multicenter retrospective study by Wen et al. in Jiangsu Province, China, found that the mean SLE Disease Activity Index (SLEDAI) score in jSLE patients was markedly higher than in both aSLE and lSLE groups (17.43 vs 16.34 vs 14.08, *P* = 0.031). Additionally, jSLE patients had a higher incidence of butterfly rash (76.1%) and proteinuria (54.5%). They also demonstrated notably higher rates of anti-dsDNA antibody positivity and greater complement C3/C4 consumption (12).

The clinical features of aSLE generally fall between those of jSLE and lSLE. A study by Mongkolchaiarunya et al. showed that aSLE patients presented with malar rash, arthritis, leukopenia, and lymphopenia more often than lSLE patients, and they also exhibited slightly higher disease activity. The incidence of nephritis is significantly higher in aSLE patients than in lSLE patients (74.2% vs 53.2%, *P* = 0.008) (14). Furthermore, complement activation products (such as C3a and C5a) and autoantibodies (such as anti-dsDNA) are considered noninvasive biomarkers for monitoring disease activity and predicting relapse. These biomarkers may be more pronounced in eSLE patients (18).

In contrast, lSLE typically has a more insidious clinical onset. These patients tend to have lower rates of positive immunological antibodies and distinctive patterns of organ involvement compared to younger SLE patients (19). A systematic review by Medlin et al. indicated that lSLE patients had notably lower odds of developing malar rash (odds ratio [OR] = 0.43), photosensitivity (OR = 0.72), and Raynaud’s phenomenon (OR = 0.84) compared to aSLE patients (20). lSLE patients are also more likely to experience pulmonary involvement, with the risk of interstitial lung disease (ILD) approximately 2.56 times higher than in eSLE (OR: 2.56; 95% confidence interval [CI]: 1.27–5.16) (21). Additionally, a study by Riveros-Frutos et al., using the Spanish RELESSER registry (3,619 patients) found that lSLE patients more frequently developed serositis, thrombotic events, severe depression, and cardiovascular diseases, and had positive lupus anticoagulant values. That study also noted that lSLE patients had a higher organ damage index (SDI) (15).

In summary, SLE patients of different ages show significant clinical and immunological heterogeneity. Age at onset is not only a fundamental factor underlying this heterogeneity, but should also be considered a critical stratification variable when developing individualized diagnostic and therapeutic strategies (**Table 1**).

**Table 1:** Age-related differences in clinical and immunological characteristics of SLE patients.

Age group	Disease phenotype	Immunological characteristics	Organ involvement	Disease progression	References
jSLE(<18 years)	Acute, severe onset; High disease activity	Strongest serology; Marked hypocomplementemia; Strong immune activation;	High prevalence of nephritis, Neuropsychiatric SLE, Hematologic manifestations	Aggressive course; Early organ damage; Higher mortality if untreated	(12, 17, 22, 23)
aSLE(18–50 years)	Variable disease activity; Heterogeneous presentation	Mixed antibody profile; High autoantibody titers; Low complement levels	Multi-organ involvement common (renal, skin, joints, CNS)	Intermediate severity; Better short-term response; Frequent relapses; Variable long-term outcomes	(14, 18, 24)
lSLE(≥50 years)	Insidious onset; Lower disease activity at baseline	Low autoantibody titers; Mildly reduced complement; Weaker immune activation	More musculoskeletal and serosal involvement; Less cutaneous and renal at onset; Higher comorbidities	Slower disease progression but higher risk of chronic organ damage and mortality	(14, 15, 19, 20)

## Clinical heterogeneity of LN and age-related characteristics

Significant differences exist in the clinical manifestations, renal pathology, and disease progression of LN among SLE patients from different age groups.

jSLE patients are considered a high-risk group for LN; indeed, the majority develop LN within two years of their SLE diagnosis (25). In LN patients with childhood-onset SLE, the immune-inflammatory response is often more intense and renal function deteriorates more rapidly. Clinically, such patients often present with heavy proteinuria, hypocomplementemia, and high-titer anti-dsDNA antibodies. Kidney biopsies in this group more frequently reveal proliferative changes (Class III or IV), underscoring the need for early identification and intervention (26, 27). A retrospective study of Asian jSLE patients found that younger-onset cases were more likely to develop renal involvement at an earlier stage; more than half had kidney involvement at the time of SLE diagnosis. Moreover, prompt and adequate treatment substantially reduced the incidence of ESRD in this cohort (28). In addition to immune responses, metabolic abnormalities-such as alterations in bone mineral content and dyslipidemia-may indirectly influence the progression of LN in SLE patients by amplifying inflammatory activity and promoting structural renal damage (29, 30).

Clinical and renal pathological manifestations in aSLE vary widely, ranging from highly active proliferative lesions (Class III/IV) to Class V membranous and mixed lesions (31). Studies indicate that the median age of progression to LN in aSLE patients is around 31.4 years (32). Compared to lSLE patients, those with aSLE are more likely to achieve complete renal response (CR) at 6 and 12 months with standard immunosuppressive therapy, and they generally have a better short-term prognosis. However, aSLE is also characterized by greater disease fluctuation and a higher relapse rate, underscoring the need for long-term, standardized follow-up (24).

LN in lSLE patients typically has a relatively insidious presentation. Kidney damage progresses more slowly in this group. However, because of lower immune activity, their autoantibody levels and disease activity indices tend to be lower, and multiple age-related comorbidities are often present. These factors can lead to misdiagnosis or delayed diagnosis (33). Despite relatively mild initial clinical manifestations, elderly patients with LN often exhibit more severe chronic lesions on renal pathology. Additionally, due to poor tolerance of intensive immunosuppressive therapy, the risk of long-term renal deterioration in this group remains significant (13, 34). A long-term follow-up study by Calatroni et al. (260 LN patients) found that late-onset patients had a considerably higher chronic damage index at disease onset compared to jSLE and aSLE patients, and late-onset disease was an independent predictor of progression to chronic kidney disease (CKD) or death (35). In particular, kidney structural alterations driven by cellular senescence, such as renal fibrosis, represent a central pathological basis for irreversible renal functional decline (36). This chronic pro-inflammatory and pro-fibrotic process occurs independently of traditional immune complex deposition, which partly explains why intensified immunosuppressive therapy alone often yields limited efficacy in elderly LN patients and fails to fully restore renal function.

Pediatric-onset LN is characterized by intense inflammation, extensive damage, and rapid progression. Adolescent-onset LN tends to be highly active and responds well to treatment, but is marked by frequent disease flares. By contrast, LN in older patients may have relatively mild immune marker levels yet is more likely to lead to poor long-term outcomes due to metabolic factors and chronic structural damage (**Table 2**). Developing age-specific assessment and intervention strategies is expected to enhance early detection of LN and improve long-term renal outcomes.

**Table 2:** Clinical heterogeneity and age-related characteristics of LN patients.

Age group	Clinical features	Renal pathology	Treatment response	Prognosis	References
jSLE(<18 years)	Early LN onset (often within 2 years), heavy proteinuria, hypertension, rapid renal decline	More proliferative lesions (Class III/IV)	Generally sensitive to immunosuppressive therapy, but frequent relapses	Aggressive course, higher risk of ESRD if untreated	(25–28)
aSLE(18–50 years)	Heterogeneous presentation, variable renal involvement	Wide spectrum: Class III/IV to Class V	Higher CR rates at 6-12 months under standard therapy, but higher relapse risk	Better short-term outcome, long-term relapse remains a challenge	(24, 31)
lSLE(≥50 years)	Insidious onset, often misdiagnosed/delayed, slower renal progression	More chronic lesions (fibrosis, tubular atrophy)	Poor tolerance to intensive therapy, lower remission rates	Worse long-term outcome due to CKD progression and comorbidities	(13, 33, 34, 36)

## Age-related risk factors for the progression of SLE to LN

The mechanisms underlying the progression of SLE to LN are complex, involving immunological, genetic, inflammatory, and clinical factors. Several classic immunological markers are key risk indicators: positive anti-dsDNA antibodies, low complement C3/C4 levels, and the presence of anti-C1q antibodies all reflect immune complex-mediated damage and are associated with a higher risk of developing LN (37). Additionally, elevated pro-inflammatory cytokines (e.g., IL-6, IFN-α), renal tubular injury markers (e.g., urinary NGAL, MCP-1, TWEAK), and urinary abnormalities (e.g., subclinical proteinuria, microscopic hematuria) have been closely linked to the early onset of LN (38–40). Moreover, clinical features such as hypertension, hypoalbuminemia, and thrombocytopenia are confirmed high-risk factors for LN (41, 42). At the genetic level, variants in genes like STAT4 (rs11889341), ADD2, and NCX1, as well as a weighted genetic risk score encompassing 112 non-HLA and HLA-DRB1 loci, are strongly associated with the development of LN (43–45). These genetic markers provide a basis for early identification and stratified management of high-risk individuals.

Analyzing LN risk from an age-stratified perspective shows that the susceptibility to LN is governed by a combination of immune status, metabolic comorbidities, and cellular senescence mechanisms that differ by ages of disease onset. Adolescent SLE patients are typically in a state of heightened immune activity, often marked by high anti-dsDNA titers, persistent complement C3/C4 consumption, and overexpression of pro-inflammatory cytokines (e.g., IL-6, IFN-α) (40, 46). Correspondingly, urinary biomarkers such as TWEAK, MCP-1, and NGAL tend to rise early in this group, potentially serving as preclinical indicators of renal involvement (47). In contrast, LN risk factors in elderly SLE patients depend more on metabolic burden and an imbalance of immune homeostasis. Comorbidities like hypertension, diabetes, hypoalbuminemia, and dyslipidemia are more prevalent in older patients. These conditions lead to glomerular filtration dysfunction and impaired microcirculation, thereby promoting tubular-interstitial damage and chronic fibrosis (41, 48). This metabolic vulnerability, combined with immunesenescence leading to impaired cellular function (e.g., reduced ability to clear immune complexes), constitutes the basis for the chronic progression of LN and the accumulation of structural damage in older patients (49, 50).

Recent studies have provided statistical evidence that patient age is an independent predictor for progression to LN. For example, the RIFLE-LN risk model developed by Chan et al. (based on 1,652 SLE patients) identified younger age at onset, positive anti-dsDNA antibodies, and male sex as independent predictors of LN, with the model showing good validation performance (AUC = 0.70) (51). Similarly, a retrospective cohort study by Katechis et al. found that an onset age below 26 years was associated with a 3.71-fold higher risk of developing LN compared to onset at 26 years or older (adjusted HR: 3.71; 95% CI: 1.84–7.48). This effect remained significant even after adjusting for sex, autoantibody status, and other clinical factors (46). Another set of studies emphasized that elderly SLE patients experienced a more rapid decline in estimated glomerular filtration rate (eGFR) during follow-up, suggesting that age may not only be a predisposing factor for LN but also contribute to renal function deterioration later in the disease process by influencing treatment response and renal reserve capacity (52).

## Current status of age-related LN prediction models and assessment tools

With a deeper understanding of LN pathogenesis and individual variability, predictive models have taken on an increasingly important role in clinical risk assessment and early intervention. In recent years, several research teams have developed LN risk prediction models based on SLE patient cohorts. These models employ techniques ranging from logistic regression and Cox proportional hazards analysis to LASSO regression and ensemble learning (e.g., random forest, XGBoost), aiming to identify high-risk patients likely to develop LN early in the disease course (8, 53). **Table 3** summarizes the main findings of some of the prediction models.

**Table 3:** Summary of the predictive model.

References	Method	Predictors	Reported performance	External validation	Age-stratified analysis	Practical notes
Tang et al. (38)	LASSO-logistic regression analysis	Erythrocyte sedimentation rate, Mucosal ulcer, Proteinuria, Hematuria	AUC=0.711	No	No	Moderate discrimination
Shin et al. (44)	Logistic regression analyses	Onset age, Pleuritis, Pericarditis, Anti-dsDNA antibodies, Anti-Smith antibodies, Genetic risk score	No performance metrics reported	No	No(age was included only as a continuous variable)	Cannot evaluate discrimination; Treatment data not accounted for
Katechis et al. (46)	Multivariate Cox proportional hazards model	Onset age, Sex, Anti-dsDNA antibodies	AUC=0.724 (Validation cohort)	Yes	Yes(< 26 years old,≥26 years old)	High discrimination; Limited generalizability, Untreated confounding (therapy)
Feng et al. (48)	Logistic regression analyses	Albumin, Uric acid, Total cholesterol	AUCs for complete models integrating multiple factors were not reported	No	No	Incorporated metabolic markers, but lacked comprehensive prediction mode
Chan et al. (51)	Cox regression analysis	Onset age, Sex, Anti-dsDNA, SLE duration	AUC=0.70(test set); Sens=0.73; Spec=0.57	Yes	Yes(< 18 years old, 18-50 years old, > 50 years old)	Moderate discrimination; Limited generalizability beyond Chinese population
McDonald et al. (53)	LASSO logistic regression	Age, haemoglobin, Baseline damage, 24-hour urine protein, Active lupus in haematological and mucocutaneous domains	AUROCs: 0.56(improvement), 0.55 (CR), 0.51 (PR)	No	No(age was included only as a continuous variable)	Weak discrimination; Trial-based data

Most models use clinical characteristicsd, immunological and urinary parameters (anti-dsDNA antibodies, complement C3/C4, proteinuria and hematuria) as predictors, and many include age as a covariate. However, the statistical performance and validation of these models vary substantially. For example, the RIFLE-LN risk score (Chan et al.)-derived from a territory-wide longitudinal cohort and tested in an independent testing set (n=270)-yielded an AUC of 0.70 with sensitivity 0.73 and specificity 0.57 in the testing cohort, illustrating moderate discriminatory ability but modest specificity in that population (51). Analyses based on trial data highlight additional caveats. A LASSO analysis of the ALMS induction cohort (n = 370) found that older age was associated with higher odds of improvement at 6 months (OR: 1.03 per year) (53). Given the discrepancy between this finding and other studies, further research is needed to clarify how age influences the treatment response in LN (24, 35). Additionally, the predictive models for improvement and complete/partial renal response showed modest AUROCs (0.56, 0.55, 0.51 respectively), indicating limited discrimination for these short-term endpoints. These results illustrate that statistically significant associations do not necessarily translate into clinically useful prediction tools unless overall discrimination and calibration are adequate.

Several challenges remain in the application of LN prediction models. First, most published models lack sufficient external validation, and their generalizability to multi-ethnic, multi-center, and age-stratified populations has not been systematically assessed. Second, several models have inconsistent endpoint definitions and demonstrate weak discriminatory power for clinically important outcomes. Third, there is inadequate consideration of both non-immune factors (e.g., metabolic, structural kidney damage) and practical feasibility (e.g., test availability, cost, model interpretability). In particular, existing LN prediction models have limited applicability in specific age subgroups (e.g., pediatric or elderly patients), and there is a critical need for age-stratified evaluations.

## Conclusion and clinical translational outlook

Age at onset is a crucial clinical factor in SLE, and its role in progression to LN warrants further investigation. Current research shows that immune phenotypes, organ involvement, pathological changes, and treatment responses differ across SLE patients of different age groups. However, large- scale systematic studies are still lacking to pinpoint the independent LN risk factors and underlying mechanisms in each age group. Future research should adopt multi-center, prospective, age-stratified designs to establish a unified cohorts encompassing adolescent, adult, and elderly patients, thereby enabling systematic comparisons of LN incidence, characteristics, and prognosis across these groups.

On the mechanistic front, further studies are needed to understand the impact of immunesenescence, T/B cell reconstitution, changes in the inflammatory microenvironment, and metabolic comorbidities on LN development. This is particularly relevant to the atypical immune phenotypes seen in elderly SLE patients and their links to chronic renal progression. In addition, multi-omics approaches (e.g., single-cell sequencing, proteomics, metabolomics) should be employed to build age-specific molecular profiles of LN elucidating the potentially distinct pathways of LN development across age groups.

New risk prediction models should be tailored to the clinical and immunological profiles of SLE patients in different age groups to allow personalized risk assessment. For example, in adolescents with highly active disease, an “inflammatory-driven” model focusing on autoantibody titers and complement levels could be developed. Conversely, for older patients with less overt inflammation, an “insidious progression” tool might incorporate factors like comorbidities, chronic damage markers, and imaging findings. Furthermore, age-specific dynamic prediction systems integrating artificial intelligence could provide continuous risk updates and decision support in clinical practice.

From a translational perspective, the future goal is to establish an age-based risk stratification system for SLE-LN and to promote an integrated “early warning–intervention–monitoring” strategy. Beyond advances in mechanistic insights and methodological progress in risk prediction models, practical issues must be taken into account to enhance clinical applicability. The availability and economic burden of biomarker testing vary considerably across healthcare systems, and while high-throughput or multi-omics assays may offer additional predictive power, their widespread implementation remains limited. Furthermore, statistical performance alone does not guarantee clinical relevance; future studies should incorporate patient-centered outcomes such as long-term renal preservation, quality of life, and treatment adherence. Only by considering feasibility, cost-effectiveness, and patient needs can prediction models be successfully translated into clinical practice and improve patient outcomes. This comprehensive approach will facilitate earlier and more proactive interventions for high-risk individuals-including tailored adjustment of glucocorticoid and immunosuppressant regimens, regular renal function monitoring, and strengthened management of comorbidities-thereby advancing precision medicine and improving long-term outcomes for LN patients.
